# Gut microbial alteration in chronic spontaneous urticaria unresponsive to second generation antihistamines and its correlation with disease characteristics‐ a cross‐sectional case‐control study

**DOI:** 10.1002/clt2.70027

**Published:** 2025-01-14

**Authors:** Indrashis Podder, David Pesqué, Nerea Carrón, Pedro Iñaki González Torres, Ramon M. Pujol, Ana M. Giménez‐Arnau

**Affiliations:** ^1^ Department of Dermatology College of Medicine and Sagore Dutta Hospital Kolkata West Bengal India; ^2^ Hospital del Mar Research Institute Barcelona Spain; ^3^ Department of Dermatology Hospital del Mar Research Institute Barcelona Spain; ^4^ Universitat Autònoma de Barcelona Barcelona Spain; ^5^ Microomics Systems S.L. Barcelona Spain; ^6^ Universitat Pompeu Fabra Barcelona Spain

**Keywords:** chronic spontaneous urticaria, dysbiosis, gut, microbiome

## Abstract

**Background:**

Gut microbial involvement has been speculated in chronic spontaneous urticaria (CSU). The aim of the study was to compare the gut microbiome composition and diversity in CSU patients uncontrolled with second‐generation antihistamines (sgAHs) and healthy individuals, as well as to explore any association between gut microbiome and disease characteristics.

**Methods:**

A cross‐sectional case‐control study including 20 CSU patients unresponsive to standard doses of sgAHs, and 15 age‐and‐sex matched healthy controls was conducted. Clinico‐demographic profile, laboratory investigations and stool analysis were conducted in all study participants. 16S RNA gene sequencing and DNA isolation was performed for all stool samples, followed by bioinformatic analysis.

**Results:**

The CSU patients (mean age 39.5 ± 9.3, M:F 1:4) and healthy controls (mean age 35 ± 13, M:F 1:2) were statistically comparable. The median (IQR) duration of CSU was 42 months (7–81). Concomitant angioedema and concomitant symptomatic dermographism were present in 30% and 20% CSU patients, respectively. At inclusion, 60% patients were receiving add‐on omalizumab, while the remaining 40% were on up‐dosed sgAHs. Stool microbial analysis revealed increased diversity and higher microbial richness in CSU patients compared with healthy individuals. CSU patients showed reduced load of short‐chain fatty acid (SCFA) producing microbiota and increased load of opportunistic pathogens. The Firmicutes/Bacteroides (F/B) ratio was higher in CSU patients. Among CSU patients, higher Bacteroides and reduced Firmicutes count were associated with higher disease activity and poor control; however, there was no link with the type of therapy.

**Conclusion:**

Gut microbial dysbiosis is seen in CSU and is linked with disease control.

## 1 INTRODUCTION

Chronic spontaneous urticaria (CSU) is a common and debilitating skin disorder characterized by unpredictable onset of intensely pruritic, evanescent (<24 h) wheals with or without angioedema for more than 6 weeks, without any identifiable or definite cause. The other variant of chronic urticaria (CU) is called chronic inducible urticaria (CInDU), which can be induced by specific physical or non‐physical triggers.^1^ A recent systematic review has reported a global point prevalence of 0.7% for CU, and 2/3 of these patients are suffering from CSU.^2^

CSU is primarily a mast cell mediated disease involving bidirectional interplay between various pro‐inflammatory mediators, proteases and cytokines such as histamine, IL‐33 and bacterial products like lipopolysaccharide (LPS) and short chain fatty acids (SCFAs) including propionate and butyrate.^3^, ^4^ These bacterial products which influence mast cell activation thresholds are released by gut microbiota. Currently, IgE mediated autoallergy and IgG‐mediated autoimmunity are considered responsible for inducing mast cell activation and degranulation by acting via the FceR1 receptors.^5^ However, the exact pathogenesis of CSU remains unclear and recent researchers are hinting at additional mechanisms beyond the IgE‐IgG‐FceR1 axis. Additionally, the role of etiologic factors in CSU remains to be clarified as drugs, food, infection and systemic comorbidities may precipitate and maintain the disorder in some, while it may be idiopathic in others.

CSU exerts a considerable burden on the patients and society, adversely affecting various domains of quality of life such as sleep, work performance, sexual health, and emotional and physical well‐being.^6^ Almost one in every five CSU patients remains unresponsive to the recommended first‐ and second‐line therapies, sgAHs and add‐on omalizumab, respectively.^7^

Despite much progress in CSU related research, its exact pathogenesis and aetiology remain unclear in majority of patients. Sporadic research has postulated the involvement of both innate and adaptive immunity in CSU, reflected by altered numbers of Th2, Th17 and Treg cells both in lesional skin and serum.^8^, ^9^ Gut microbiome is a key regulator of human immune system involving both innate (toll‐like receptors on intestinal epithelium, macrophages and NK cells) and adaptive (helper/cytotoxic/regulatory T‐cells) immune pathways.^10^ The evolving relationship between gut microbiome and systemic disorders, including CSU, has become an increasingly relevant issue, because of frequent alterations in lifestyle, climate and dietary habits, which are important influencing factors.^11^ However, a possible relationship between gut microbiome and CSU has been sparsely evaluated.

In this context, we aimed to investigate the gut microbiome composition and diversity in CSU patients refractory to sgAHs compared with healthy controls, as well as to explore the relationship between gut microbiome and the clinical (concomitant symptomatic dermographism, angioedema), laboratory (absolute eosinophil and basophil counts, serum IgE concentration and basophil FceR1 receptor density) and disease course (disease duration, disease severity, disease control) in the subset of CSU patients.

## 2 METHODS

### 2.1 Study characteristics and participants; inclusion criteria

We conducted a cross‐sectional case‐control pilot study including 20 patients with CSU and 15 age‐and‐sex matched healthy controls. Requisite permission was obtained from the Institutional Ethics committee (*2022/10507/I*). Criteria for inclusion in the study group included adult CSU patients (≥18 years age) who failed to respond adequately to standard‐dose or up‐dosed second‐generation antihistamines (sgAHs) after 4 weeks. Inadequate response was determined by a urticaria control test (UCT) score <12. CSU was diagnosed in accordance with international guidelines. Urticaria control test (UCT) is a standardised and validated tool to assess disease control, with a score <16 indicating incomplete control, while complete control is reflected by a UCT score of 16 (maximum score).^1^ Isolated chronic inducible urticaria, presence of a known cause of urticaria like respiratory/gastrointestinal infections or parasitic infestation, hereditary angioedema, urticarial vasculitis or other urticarial syndromes, concomitant allergic disorders (atopic dermatitis, allergic rhinitis or bronchial asthma), uncontrolled systemic disorders, pregnancy or lactation, use of antibiotics, proton pump inhibitors, H2 antihistamines, pre/pro/symbiotics, and/or systemic corticosteroids within the previous 4 weeks, and denial of written informed consent comprised our exclusion criteria.

### 2.2 Evaluation of study subjects

All study participants, including cases and controls, underwent detailed history taking regarding their demographic profile. CSU patients were further enquired about their disease duration, concomitant angioedema, concomitant symptomatic dermographism or systemic comorbidities, and disease control was assessed using the UCT. We grouped CSU patients into 2 categories based on their UCT score‐ uncontrolled urticaria (UCT <12) and well‐controlled urticaria (UCT 12–16), as per current standard practice.^1^ Autologous serum skin test (ASST) was conducted in patients providing written informed consent, and they were segregated into 2 groups‐ ASST positive and ASST negative. Furthermore, we enquired about their medication status and noted CSU patients who were receiving omalizumab and/or up‐dosed sgAHs. We obtained 5 mL of venous blood from each CSU patient under aseptic conditions, and performed laboratory investigations including routine blood parameters, serum D‐dimer, serum total IgE and basophil FceR1 receptor density. Standardised biochemistry procedures were followed for these investigations viz. chemiluminescence immunoassay (CLIA) on the Elecsys system by Roche®.

### 2.3 Collection of fecal samples

Fresh fecal samples were collected from every study participant to analyse their gut microbiome. We collected approximately 1 g of fresh stool sample from every CSU patient or healthy control using a sterile swab. The fecal samples were placed in a DNA/RNA shield collection tube containing a preservative solution for genetic material. The samples were stored in a −20°C freezer, until microbiome analysis was done. Microbiome analysis was conducted at Micromics Systems S.L., Barcelona, Spain.

### 2.4 Library preparation and sequencing

DNA from samples was extracted using MagMAX CORE Nucleic Acid Purification Kit 500 RXN (Thermo Fisher, CA, Austin, USA) following the manufacturer's instructions. Samples were amplified using 16S rRNA V3‐V4 region—specific primers (V3‐V4‐Forward 5′‐TCGTCGGCAGCGTCAGATGTGTATAAGAGACAGCCTACGGGNGGCWGCAG‐3′, V3‐V4‐Reverse 5′GTCTCGTGGGCTCGGAGATGTGTATAAGAGACAGGACTACHVGGGTATCTAATCC‐3′). PCR was performed in 10‐μL final volume with 0.2‐μM primer concentration. The PCR cycle included: 3 min at 95°C (initial denaturation) followed by 25 cycles: 30 s at 95°C 30s at 55°C, and 30s at 72°C, and a final elongation step of 5 min at 72°C. PCR products were purified using AMPure XP beads (Beckman Coulter, Nyon, Switzerland) with a 0.9× ratio according to the manufacturer's instructions. The above described primers contain overhangs allowing the addition of full‐length Nextera barcoded adapters for multiplex sequencing in a second PCR step, resulting in sequencing ready libraries with approximately 450 bp insert sizes.

In brief, 5 μL of the first PCR purified product was used as template for a second PCR with Nextera XT v2 adaptor primers in a final volume of 30 μL using the same PCR mix and thermal profile as for the first PCR but with only 8 cycles. 25 μL of the second PCR product was purified with SequalPrep normalization kit (Invitrogen, ThermoFisher Scientific) according to the manufacturer's protocol. Libraries were eluted in 20 μL final volume and pooled for sequencing. Sequencing was performed using Illumina MiSeq (2 × 300 bp) and v3 chemistry with a loading concentration of 10 pM.

### 2.5 Amplicon sequences processing and analysis

Raw demultiplexed forward and reverse reads were processed using the following methods and pipelines as implemented in QIIME2 version 2020.11 with default parameters unless stated.^12^ DADA2 was used for quality filtering, denoising, pair‐end merging and amplicon sequence variant calling (ASV, i.e. phylotypes) using the qiime dada2 denoise‐paired method.^13^ Q20 was used as a quality threshold to define read sizes for trimming before merging Reads were truncated at the position when the 75th percentile Phred score felt below Q20 for forward reads and for reverse reads. ASVs were aligned using the qiime alignment mafft method.^14^ The alignment was used to create a tree and calculate phylogenetic relations between ASVs using the qiime phylogeny fasttree method.^15^ ASV tables were subsampled without replacement in order to even sample sizes for diversity analysis using the qiime diversity core‐metrics‐phylogenetic pipeline. The sample with the smallest sample size (i.e. 13,400 reads) was discarded in order to take advantage of the sequencing depth of the dataset. Subsequently, subsampling to the next lowest sample size was used for each comparison. Unweighted and weighted Unifrac distances were calculated to compare community structure.^16^

### 2.6 Statistical analysis

We tested the normality of our data using Kolmogorov–Smirnov test. Continuous variables have been expressed as mean (SD) or median (IQR), while categorical variables have been expressed as percentage, ratio or frequency. For assessing the significance of difference, we used the chi‐squared test for categorical data, *t*‐test for parametric and unpaired data, Mann–Whitney's test for non‐parametric unpaired data and paired *t*‐test for parametric paired data. In all tests, a two‐sided *p*‐value <0.05 is considered statistically significant. We will use MedCalc version 20 (Mariakerke, Belgium: MedCalc Software, 2011) software for statistical analysis and Graph‐pad Prism for graphical representation of data. Data will be preserved for future reference.

Regarding stool microbiome analysis, alpha and beta diversity analyses were performed according to the sequence results. Alpha diversity comparisons were performed using the R package NBZIMM version 1.0 for richness^17^ and the R package betareg version 3.1‐4 for evenness.^18^ Taxonomic assignment of ASVs was performed using a Bayesian Classifier trained with Silva V4 database (i.e. 99% OTUs [operational taxonomic units] database) using the qiime feature‐classifier classify‐sklearn method.^19^ Differential abundance of taxa was tested using a negative binomial linear model using the R MASS package version 7.3–58. Unifrac distance matrices and ASV tables were used to calculate principal coordinates and construct ordination plots using the R software package version 4.2.1 (http://www.R‐project.org). The significance of groups in community structure was tested using Permanova. BiodiversityR version 2.11–1 and vegan version 2.5‐5 packages were used. ggplot2 version 2.2.1 was used for plotting.

## 3 RESULTS

In the present study, both CSU patients (*n* = 20) and healthy controls (*n* = 15) were statistically comparable with respect to age (39.5 ± 9.3 years vs. 35.0 ± 13.0, *p* = 0.2), and gender (M:F 1:4 vs. M:F 1:2, *p* = 0.6). The median (IQR) duration of CSU was 42 (7–81) months. Concomitant angioedema and concomitant symptomatic dermographism were present in 30% (6/20) and 20% (4/20) CSU patients, respectively. Eleven CSU patients (55%) reported at least one co‐morbidity, the most common co‐morbidity being hypothyroidism (*n* = 5) followed by hypertension (*n* = 2). All CSU patients had moderate‐to‐severe disease activity, and the mean (SD) UAS7 score was 20.1 ± 7.1 (range 16–42). Eighteen patients (90%, *n* = 20) presented with moderate disease activity (UAS7 = 16–27), while the remaining 2 patients (10%, *n* = 20) presented with severe disease activity (UAS7 = 28–42). The mean ± SD UCT score was 8.4 ± 4.0 (range 0–13), and most patients presented with uncontrolled urticaria (UCT<12) [75% versus 25% [UCT 12–15]. ASST status could be checked in 9 patients, among them 6 (66.7%) were ASST+, while the remaining 3 (33.3%) were ASST negative. Twelve (60%) CSU patients were receiving add‐on omalizumab at the time of study, while the remaining 40% were on up‐dosed sgAHs alone (*p* = 0.3). Table 1 highlights the clinico‐demographic parameters of the study participants.

**TABLE 1:** Clinico‐demographic parameters and gut microbial analysis of study participants.

Name of parameter	CSU patients, *n* = 20	Healthy controls, *n* = 15	*p*‐value
Age in years, mean ± SD	39.5 ± 9.3	35 ± 13	0.2^a^
Gender (M:F)	4:16	5:10	0.6
Duration of CSU in months, median (IQR)	42 (7–81)	‐	‐
Angioedema, *n* (%)	6 (30)	‐	‐
Concomitant symptomatic dermographism, *n* (%)	4 (20)	‐	‐
UAS7 score, mean ± SD	20.1 ± 7.1	‐	‐
Disease activity			**0.0008** **^b^**
Moderate CSU, *n* (%)	18 (90)	‐	
Severe CSU, *n* (%)	2 (10)	‐	
UCT score, mean ± SD	8.4 ± 4	‐	‐
Disease control			**0.004** **^b^**
Uncontrolled disease, *n* (%)	15 (75)	‐	
Well‐controlled urticaria, *n* (%)	5 (25)	‐	
Current therapy			0.3
Up‐dosed sgAHs, *n* (%)	8 (40)	‐	
Up‐dosed sgAHs with add‐on omalizumab, *n* (%)	12 (60)	‐	
Gut microbiome analysis			
F/B ratio	1.43	1.33	0.2
Most abundant phyla	*Firmicutes, Verrucomicrobiota* and *Desulfobacterota*	*Bacteroidiota* and *Proteobacteria*	‐
Most abundant genera	*Weissella and Caproiciproducens*	*Weissella* and *Caproiciproducens*	‐

*Note*: Bold indicates significant difference.

Abbreviations: CSU, chronic spontaneous urticaria; F, female; F/B, *Firmicutes/Bacteroides*; IQR, inter‐quartile range; M, male; SD, standard deviation; sgAHs, second‐generation antihistamines; UAS7, urticaria activity score 7; UCT, urticaria control test.

^a^ *p*‐value obtained from independent sample *t*‐test, remaining *p*‐values obtained by Chi‐squared test.

^b^ *p* < 0.05.

Laboratory investigations revealed basopenia in 17.6% (3/17) [<20/μL], eosinopenia in 11.8% (2/17) [<20/μL], and raised D‐dimer (>500) in 31.2% (5/16) CSU patients. 27.8% (5/18) and 58.3% (7/12) CSU patients reported raised total serum IgE level (>214 IU/mL) and higher FcεR1 receptor density (>300,000 units) respectively.

**FIGURE 1:**
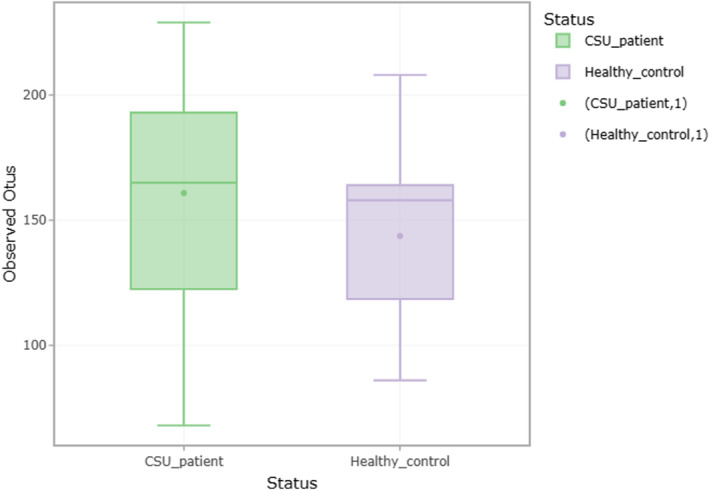
Box plots representing the distribution of operational taxonomic units (OTUs) in CSU patients (C) and healthy controls (CH). This Figure indicates a greater diversity in CSU patients.

**FIGURE 2:**
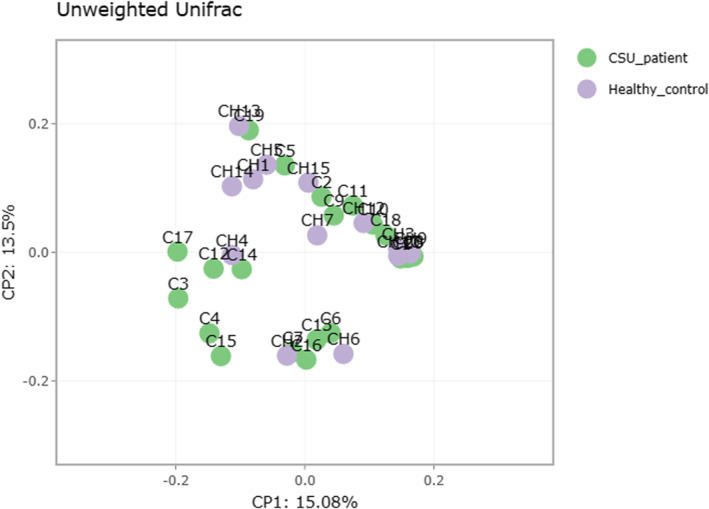
Scatter diagram showing stool microbial diversity in CSU patients (C) and healthy controls (CH).

**FIGURE 3:**
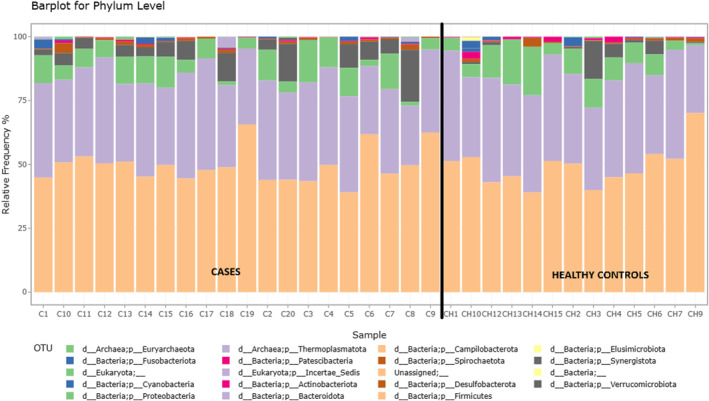
Barplot depicting the relative abundance of different microbiota in CSU patients (C) and healthy controls (CH). Each bar contains different sections, which correspond to different phylum, which can be seen in the Figure 3 legend.

Stool microbial analysis revealed no statistically significant difference in alpha‐diversity (within sample) and beta‐diversity (between samples) diversity, but diversity was greater in CSU patients (Figure 1). Overall, the number of observed OTUs (microbial richness) was higher in the CSU group compared to healthy controls (Figure 2). We obtained a total of 19 phyla in all the stool samples. In both CSU and HCs, Firmicutes was the most common phylum detected, followed by *Bacteroidota, Proteobacteria, Verrucomicrobiota* and *Desulfobacterota*. Based on relative abundance, *Firmicutes, Verrucomicrobiota* and *Desulfobacterota* were more abundant in the CSU group, whereas *Bacteroidota* and *Proteobacteria* predominated in healthy individuals. The phyla *Cyanobacteria, Eukaryota, Synergistota* and *Campilobacteriota* demonstrated almost equal distribution in both study groups (Figure 3). In our study, the F (*Firmicutes*)/B (*Bacteroidota*) ratio was higher in CSU patients compared to the healthy participants (1.43 in CSU, 1.33 in the HCs). Among these phyla, only *Actinobacteriota* showed a statistically significant difference between the groups (HC > CSU, *p* = 0.01).

We obtained a total of 227 genera in all stool samples. Among them, 160 overlapped and were detected in both CSU patients and healthy controls. The top five genera detected in both groups were *Bacteroides* (phylum Bacteroidota), *Faecalibacterium* (phylum Firmicutes), *UCG‐002* (phylum Firmicutes), *Parabacteroides* (phylum Bacteroidota) and *Alistipes* (phylum Bacteroidota). Based on relative abundance, 67 genera were differentially distributed between the two groups. Among them, *Weissella* and *Caproiciproducens*, both belonging to the phylum Firmicutes, were the most abundant genera in CSU patients and healthy controls respectively. Interestingly, at the genus level, CSU patients showed increased frequency of multiple opportunistic pathogens such as *Klebsiella, Escherichia* and *Streptococcus*, in comparison to healthy controls.

**FIGURE 4:**
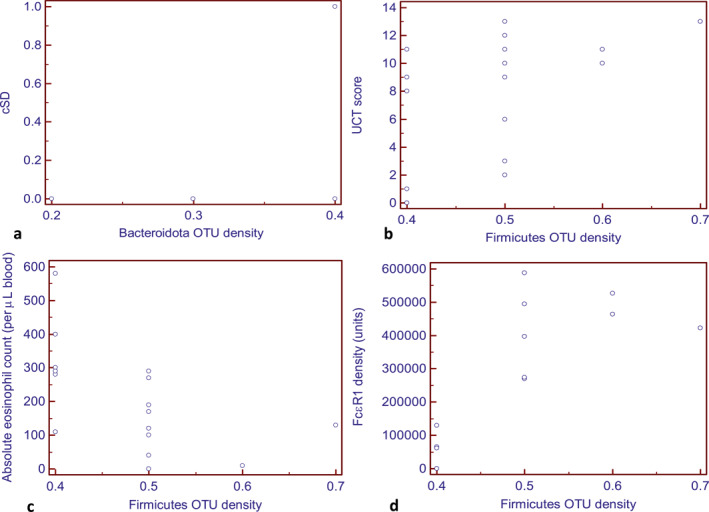
Scatter diagram showing significant correlation between (A) Concomitant symptomatic dermographism (cSD) and Bacteroidota, (B) UCT (C) Absolute eosinophil count and (D) FceR1 density with Firmicutes.

Intra‐group analysis (CSU patients, *n* = 20), revealed a significant positive correlation between concomitant symptomatic dermographism and *Bacteroidota* OTU density (*ρ* = 0.5, *p* = 0.03), UCT score and *Firmicutes* (*ρ* = 0.4, *p* = 0.04), and basophil FcεR1 receptor density and *Firmicutes* (*ρ* = 0.7, *p* = 0.006) (Figure 4). Absolute eosinophil count showed a significant negative correlation with *Firmicutes* (*ρ* = −0.6, *p* = 0.02) (Figure 4). *Actinobacteroita* and *Firmicutes* showed a negative relationship with all disease parameters of CSU, except disease control and basophil FceR1 receptor density. Table 2 summarizes the relationship between gut microbiota and CSU disease characteristics. Interestingly, the gut microbial composition did not seem to be affected by the therapy received by the patients (up‐dosed sgAHs or omalizumab).

**TABLE 2:** Correlation between gut microbiota and disease characteristics in CSU patients (*n* = 20).

Name of gut microbiota	Disease characteristics																	
	Duration		Disease severity		Disease control		cSD		AO		AEC		ABC		Serum IgE		Basophil FceR1 receptor density	
	*r*	*p*	*r*	*p*	*r*	*p*	*r*	*p*	*r*	*p*	*r*	*p*	*r*	*p*	*r*	*p*	*r*	*p*
VERRUCOMICRO	−0.2	0.5	0.08	0.7	−0.4	0.1	−0.3	0.1	0.3	0.2	0.2	0.5	0.2	0.4	−0.06	0.8	−0.2	0.5
ACTINOBACTEROITA	−0.03	0.9	−0.1	0.6	0.2	0.4	−0.1	0.6	−0.1	0.5	−0.2	0.5	−0.1	0.6	−0.2	0.5	0	1
BACTEROIDOTA	0.4	0.1	0.3	0.1	−0.01	0.9	**0.5**	0.03*	−0.07	0.7	0.3	0.2	−0.05	0.8	0.1	0.7	−0.3	0.3
EURYARCHAEOTA	0.05	0.8	0.06	0.8	−0.1	0.5	−0.1	0.6	0.3	0.1	0.1	0.6	0.03	0.9	−0.1	0.5	0.1	0.7
PROTEOBACTERIA	0.2	0.3	0.2	0.4	−0.1	0.6	0.2	0.3	0.05	0.8	0.3	0.2	−0.1	0.6	0.07	0.8	−0.6	0.06
DESULFOBACT	−0.05	0.8	0.1	0.6	−0.2	0.5	−0.2	0.5	0.2	0.4	−0.05	0.8	0.009	0.9	−0.3	0.2	−0.2	0.6
FIRMICUTES	−0.3	0.2	−0.4	0.06	**0.4**	0.04*	−0.2	0.4	−0.3	0.2	**−0.6**	0.02*	−0.1	0.6	−0.2	0.5	**0.7**	0.006*

*Note*: ‘−’ sign indicates negative correlation, * indicates statistical significance (*p* < 0.05), bold indicates significant difference.

Abbreviations: ABC, absolute basophil count; AEC, absolute eosinophil count; AO, angioedema; cSD, concomitant symptomatic dermographism; IgE, immunoglobulin E; r, ‘rho’ value (correlation coefficient).

## 4 DISCUSSION

Gut microbiome, an emerging area of medical research, is responsible for maintaining human health by regulating both innate and adaptive immunity.^10^ The term ‘microbiota’ refers to all microorganisms residing inside the human body including bacteria, fungi, and prokaryotes, while the genetic material of these organisms constitute ‘microbiome’.^20^ The most predominant species in the human gastrointestinal tract are *Firmicutes and Bacteroidetes* sp.^21^ These microorganisms release SCFAs which form the backbone of gut‐skin axis by multiple mechanisms such as regulating the growth and virulence of pathogenic gut microbes like *Escherichia coli* and *Klebsiella sp.,* ^22^ maintaining the intestinal epithelial integrity by upregulating tight junction molecules, thereby preventing the release of toxic LPS into blood stream,^23^ and inducing the release of anti‐inflammatory cytokine interleukin (IL)‐10, and prostaglandin (PG)E2.^24^ Additionally, beneficial bacteria secrete several vitamins and amino acids, further influencing the immune system.^25^ In the context of urticaria, LPS may facilitate mast cell degranulation via Toll‐like receptor 4 (TLR4) resulting in the release of pro‐inflammatory cytokines, while SCFAs may inhibit both IgE‐mediated and non‐IgE‐mediated mast cell activation via anti‐inflammatory Il‐10 and PGE2.^26^ To date, there is no standard definition of a healthy gut microbiome, but important characteristics include high levels of diversity, stability, resistance to stress and a high level of redundancy of metabolic pathways.^27^ Normal human microbiota is composed of commensal bacteria which primarily includes indigenous symbiotic bacteria.^28^

Qualitative or quantitative gut microbial alterations, called dysbiosis, have been linked to several inflammatory dermatoses such as acne, rosacea, psoriasis, food allergies and atopic dermatitis owing to their immune regulatory role.^29^, ^30^ Interestingly, there is an emerging body of evidence linking gut microbial dysbiosis and chronic spontaneous urticaria; however, its relationship with disease characteristics remains poorly understood.^4^, ^10^, ^20^, ^24^, ^31^ The proposed mechanisms include reduced gut microbial diversity, reduced levels of anti‐inflammatory SCFAs, and therapeutic benefit of probiotic supplements in some patients; however, the precise pathogenic role and relevance of gut microbiome in CSU remain poorly characterized.^24^

In the present study, no statistically significant differences were detected in terms of alpha (single sample) and beta (ratio of operational taxonomic units or OTUs between samples) diversity between CSU patients and healthy individuals. However, CSU patients demonstrated a higher number of OTUs (vs. healthy controls), thus indicating a disruption of gut microbiota. Other authors have also detected marked microbiome variation in CSU subjects, compared to healthy controls, with significant beta diversity^20^ or alpha‐diversity.^24^

The top five phyla detected in all stool samples in our study were Firmicutes, Bacteroidota, Proteobacteria, Verrucomicrobiota and Desulfobacterota. This result is in agreement with previous studies which have shown that these phyla constitute the majority of human intestinal microbiota, with the two phyla Firmicutes and Bacteriodiota, representing 90% of the gut microbiota.^32^, ^33^

Our CSU patients showed reduced load of *Bacteroidota* in the gut compared to healthy controls, which is in agreement with previous studies.^10^, ^34^, ^35^, ^36^ *Bacteroidota* are the principal producers of short‐chain fatty acids (SCFAs e.g. butyrate, isobutyrate, isocaproate, and caproate), which downregulate the immune system by inducing regulatory T cell differentiation, enhancing IL‐10 production and inhibiting Th17 cells.^37^ Thus, reduced concentrations of anti‐inflammatory *Bacteroidota* in CSU patients may explain the persistent inflammatory state in CSU and its link to gut microbiome. At the family level, our CSU patients showed reduced levels of *Rikenellacaea* and *Roseburia*, major producers of SCFA, similar to Zhu et al.^24^ Nabizadeh et al.^35^ detected higher prevalence of SCFA producing Bifidobacterium and Lactobacillus in healthy individuals compared to CSU patients. In comparison, we found higher levels of Bifidobacterium in healthy individuals, but Lactobacillus was raised in CSU patients, consistent with Yuksekal et al.^20^ The beneficial role of SCFAs in CSU is further corroborated by reports of improvement in disease severity by administration of probiotics containing SCFA‐producing bacteria such as *Lactobacillus* or *Lactobacillus* combined with *Bifidobacterium* to such patients.^38^, ^39^

Our study showed an increased population of *Firmicutes* phylum in CSU patients, consistent with Zhang et al.^31^ *Firmicutes* are beneficial bacteria responsible for breaking insoluble fibres, and their relevance in CSU remains undetermined. In our study, *Actinobacteriota* (syn *Actinobacteria*) were significantly higher in controls, which may be attributed to their pivotal role in maintaining gut homeostasis.^40^ Opportunistic pathogens such as *Klebsiella*, *Escherichia* and *Streptococcus* were more abundant in the CSU group, compared to healthy controls, consistent with other authors.^24^, ^35^ *Klebsiella* derived lipopolysaccharide (LPS) may exacerbate mast cell driven skin inflammation as recently observed in a mouse‐model study.^24^

Currently, Firmicutes/Bacteroidota (F/B) ratio is considered to reflect the general composition of intestinal microbiota as it involves the two most abundant phyla. A higher ratio has been detected in several inflammatory and neoplastic disorders including obesity, diabetes, colorectal carcinoma and osteosarcoma.^41^ Our study also showed an increased F/B ratio in CSU patients compared with healthy controls (1.43 vs. 1.33), corroborating its role as an inflammatory marker. In contrast, Yuksekal et al.^20^ and Bai et al.^42^ reported lower F/B ratios in CSU and diabetic retinopathy patients compared with healthy controls. Notably, the ideal F/B ratio is yet to be established in healthy individuals, so both low and high values may be associated with systemic inflammatory disorders.

Intra‐group analysis of the CSU patients revealed a significant positive correlation between *Bacteroidota* and concomitant symptomatic dermographism, *Firmicutes* and UCT score, and *Firmicutes* and basophil FcεRI receptor density; whereas *Firmicutes* also demonstrated a negative correlation with eosinophil count. Therefore, the predominance of *Bacteroidota* and reduced *Firmicutes* may be considered markers of disease activity and poor disease control. However, large‐scale studies are needed to validate this claim. Notably, Liu et al. reported a negative correlation between *Subdoligranulum* and *Ruminococcus bromii*, and concomitant symptomatic dermographism.^36^ The composition of the gut microbiota remained unaffected by the type of treatment used, antihistamines or omalizumab, reflecting the non‐interference of both therapies in the gut milieu. The role of antihistamines in altering gut microbiome in CSU patients (vs. healthy controls) has been speculated and needs further exploration.^43^

This study has strengths and limitations to enhance. It has been performed in a real‐life setting including CSU patients refractory to standard‐doses of sgAHs. However, limitations to be considered are the small sample size, inability to evaluate the faecal pH, and the presence of potential confounding uncontrolled factors such as dietary habits, height, weight and environmental influences.

## 5 CONCLUSION

In this study, the F/B ratio was higher in CSU patients than in healthy individuals, suggesting gut microbial dysbiosis in CSU. At the genus level, opportunistic pathogens were also more abundant in the CSU group. *Bacteroidota* and *Firmicutes* may be linked to increased disease activity and poor disease control. The gut microbiome composition was independent of the type of treatment, antihistamines or omalizumab. All these data indicate a possible association between dysbiosis and CSU, and its potential role as a predictor of disease severity and activity. The debatable relationship between diet and CSU may be further explored in the light of gut microbiome as dietary habits are known to affect the microbial milieu. However, further large‐scale, multicentric, control‐matched studies will be needed to clarify the exact role of gut microbiome in CSU. The role of gut microbiome as a possible marker for therapeutic efficacy may also be explored in the near future.

## AUTHOR CONTRIBUTIONS

**Indrashis Podder**: Conceptualization; Investigation; Funding acquisition; Writing ‐ original draft; Writing ‐ review and editing. **David Pesqué**: Investigation; Writing ‐ original draft; Writing ‐ review and editing. **Nerea Carrón**: Methodology; Validation; Visualization; Software; Formal analysis; Data curation. **Pedro Iñaki González Torres**: Methodology; Validation; Visualization; Software; Formal analysis; Data curation. **Ramon M. Pujol**: Supervision; Writing ‐ review and editing; Project administration. **Ana M. Giménez‐Arnau**: Project administration; Supervision; Writing ‐ review and editing.

## CONFLICT OF INTEREST STATEMENT

Indrashis Podder has no conflicts of interest in relation to the present report. Outside of it, he is or recently was a speaker and/or advisor for Menarini, Sun Pharmaceuticals, Glenmark India and Alkem Laboratories. Ana Maria Gimenez‐Arnau has no conflicts of interest in relation to the present report. Outside of it, she is the Medical Advisor for Uriach Pharma/Neucor, Genentech, Novartis, FAES, GSK, Sanofi–Regeneron, Amgen, Thermo Fisher Scientific, Almirall, Celldex, Leo Pharma Research Grants supported by Uriach Pharma, Novartis, Grants from Instituto Carlos III‐ FEDER Educational activities for Uriach Pharma, Novartis, Genentech, Menarini, LEO‐PHARMA, GSK, MSD, Almirall, Sanofi, Avene. All other authors declare no conflicts of interest in relation to the present study.

## Data Availability

Further data, if necessary, would be available from the corresponding author upon reasonable request.
